# Robot‐Assisted Radical Prostatectomy Using the Hugo RAS System: The First 15 Cases by a First‐Year Novice Surgeon Without Prior Console Experiences

**DOI:** 10.1002/rcs.70249

**Published:** 2026-11-02

**Authors:** Che‐Hsueh Yang, Daniela‐Viviana Barbulescu, Lucian Marian, Yuriy Aleksandrovich Kim, Chi‐Hsiang Wu, Ragavan Narasimhan, Yen‐Chuan Ou, Chi‐Chien Lin

**Affiliations:** ^1^ Department of Urology Chang Bing Show Chwan Memorial Hospital Changhua Taiwan; ^2^ Doctoral Program in Translational Medicine National Chung Hsing University Taichung Taiwan; ^3^ Rong Hsing Translational Medicine Research Center National Chung Hsing University Taichung Taiwan; ^4^ Department of Medical Oncology ONCOHELP Hospital Timisoara Romania; ^5^ Department of Urology “Pius Brînzeu” County Emergency Clinical Hospital Timisoara Romania; ^6^ Department of Urology Botkin Hospital Moscow Russia; ^7^ Departments of Urology Apollo Hospitals Chennai India; ^8^ Department of Surgery Division of Urology Tungs' Taichung MetroHarbor Hospital Taichung Taiwan; ^9^ Institute of Biomedical Science The iEGG and Animal Biotechnology Center National Chung Hsing University Taichung Taiwan

**Keywords:** blood loss, confidence interval, learning curve, nadir, prostate cancer, prostatectomy, surgery, urinary continence, urinary system

## Abstract

**Background:**

No study has specifically addressed the initial supervised experience of novice surgeons using the Hugo RAS system.

**Methods:**

We retrospectively reviewed patients with prostate cancer receiving robot‐assisted radical prostatectomy (RARP). Console time and blood loss were used as intraoperative variables, while oncological and functional outcomes were evaluated postoperatively.

**Results:**

In the first 15 cases, both console time and blood loss showed a trend towards improvement as the novice surgeon gained experience using the Hugo RAS system for RARP. Regarding oncological and functional outcomes, 80% of patients achieved an undetectable PSA nadir, and 80% recovered urinary continence within 6 months after surgery.

**Conclusion:**

Hugo RAS–assisted RARP was feasible during this supervised novice‐surgeon experience, with early adaptation in console time and blood loss. However, larger prospective studies with standardized outcome measures are needed to validate these findings.

## 1 Introduction

In the literature, radical prostatectomy (RP) was one of the earliest surgeries performed using a robotic system, and robot‐assisted RP (RARP) has been evolving for 25 years [1]. Although robotic platforms were not widespread initially, and few urologists had expertise in RARP in the past [2], nowadays, approximately 90% of RPs are performed using robotic platforms [3]. RARP offers several advantages over laparoscopic RP, such as eliminating the need for complex laparoscopic surgical skills and having a shorter learning curve [4]. Among robotic platforms, the da Vinci (DV) robotic surgical system (Intuitive Surgical, Sunnyvale, CA, USA) once monopolised the global market. Revenue from robotic surgery has continued to grow, remaining unaffected even during the COVID‐19 pandemic [5]. To make matters even better, high medical costs have begun to decrease as several worthy competitors have entered the industry. With this trend, RARP will likely continue to thrive as a mainstream treatment option for localised prostate cancer (PCa). According to the literature [6], at least 10 robotic systems, including the DV system, are available worldwide.

The Hugo RAS system (Medtronic, Minneapolis, MN, USA) is the most cost‐effective and most discussed novel multiport robotic platforms [6]. Among the published reports on surgical procedures performed using the Hugo RAS system, RARP remained the most extensively studied, with 19 published articles [6]. Based on the current literature, RARP performed using the Hugo RAS system appears to be associated with longer docking and console times than the DV system. However, estimated blood loss is not significantly increased [6]. Furthermore, no significant differences have been observed between the two platforms regarding positive surgical margins, PSA status at 3 months, or urinary continence at 3 months. Nevertheless, the available evidence is characterised by substantial heterogeneity in study design [6], surgeon experience, patient selection, and outcome reporting, making it difficult to draw definitive conclusions regarding the comparative performance of the Hugo RAS and DV system. The first prospective study conducted in the United States reported findings consistent with previous studies, particularly regarding the longer docking time associated with the Hugo RAS system [6, 7]. Although one patient experienced a grade V complication, the fatal event was determined to be unrelated to the Hugo RAS system [7].

Despite the heterogeneity among the available studies, the question of greatest interest to urologists is whether the surgical experience and techniques developed with the DV system can be effectively translated to the Hugo RAS system. Regarding neurovascular bundle preservation, early evidence suggests that meticulous nerve‐sparing techniques are feasible with the Hugo RAS system and have demonstrated promising preliminary outcomes [8].

In our previous experience with the Hugo RAS system, no conversions were observed among the initial 30 cases [9]. RARP performed by an experienced surgeon can facilitate rapid adaptation to the Hugo RAS system. Although certain surgical steps may take longer, the overall console time was not significantly different from that of the DV system [9]. Similar findings have been reported in other published studies, including the first comparative study conducted in Asia [10]. However, both of these pioneering studies were performed by experienced surgeons. Based on these findings, we further investigated RARP performed using the Hugo RAS system by a first‐year novice surgeon with no prior console experience in robotic surgery and reported our experience with the first 15 cases.

## 2 Materials and Methods

### 2.1 Study Design and Population

Between November 1, 2024, and July 1, 2025, men with PCa who underwent RARP using the Hugo RAS system were reviewed following approval from the Institutional Review Board at Chang Bing Show Chwan Memorial Hospital (Changhua, Taiwan; IRB: SCMH_IRB.No 1150109). The ethics committee approved this review of medical files and all steps followed the ethical rules of the Declaration of Helsinki. The PCa was all diagnosed by transrectal ultrasound‐guided prostate biopsy (TRUSP), and RARP was arranged between 1 and 4 weeks after TRUSP. Magnetic resonance imaging (MRI) was performed in all patients diagnosed with PCa before surgery. A whole‐body bone scan was additionally performed in patients with a risk category of intermediate unfavourable or higher (National Comprehensive Cancer Network. Prostate Cancer (Version 2.2025), Plymouth Meeting, PA, USA. URL: https://www.nccn.org/guidelines/guidelines‐detail?id=1459).

### 2.2 Surgeon Experience and Training

All RARP procedures were performed by Che‐Huseh Yang, MD (Figure 1a), who had no prior console experience in robotic surgery on any platform. Before becoming an attending physician, he had assisted as a bedside assistant in more than 300 RARP cases with the DV system during his residency. He had no prior experience with open RP and had assisted in only two cases of laparoscopic RP. Che‐Hsueh Yang, MD, received dry‐lab training on April 28 and 29, 2023, and served as a bedside assistant in the animal lab on April 30, 2023. The dry lab and animal lab were held by Medtronic (Minneapolis, MN, USA) at IRCAD (Lukang, Changhua, Taiwan) and Ragavan Narasimhan, MD [10], serving as the instructor. Certification was granted upon completion of the training; however, no surgical procedures were observed after the dry‐lab and animal‐lab training. Between March 1 and August 31, 2023, he assisted in 30 RARP cases performed by Yen‐Chuan Ou, MD [5, 9], serving as a bedside assistant using the Hugo RAS system. Before performing his first RARP procedure using the Hugo RAS system, he practiced operating the console controls using the built‐in simulator for 4 hours.

**FIGURE 1:**
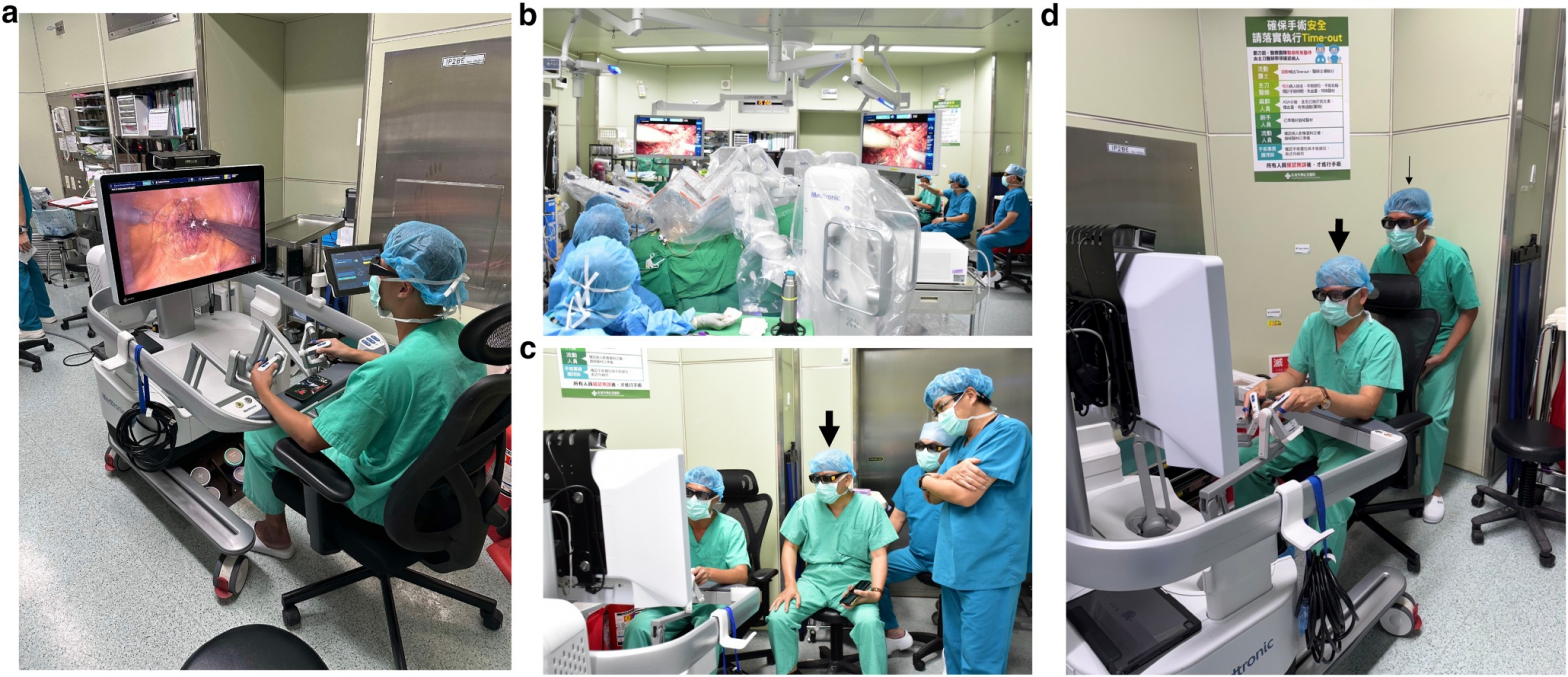
(a) The surgeon operating through the console cart. (b) The operating room environment. (c) The instructor (arrow) sat next to the operator for providing assistance. (d) The instructor (thick arrow) provided the assistant when the operator (thin arrow) called for it.

### 2.3 Surgical Techniques

The surgical steps followed the same protocol as described in previous publications [5, 9]. Before performing RARP, the patients would be assessed using the online nomogram (Prostate cancer nomograms (Pre‐Radical Prostatectomy), Memorial Sloan Kettering Cancer Centre, New York, NY, USA. URL: https://www.mskcc.org/nomograms/prostate/pre_op) regarding the survival prediction and lymph node involvement probability. Pelvic lymph node dissection was performed during RARP when the predicted probability of lymph node involvement exceeded 2%. The lymph node dissection included the bilateral obturator fossae and the bilateral external iliac regions.

Regarding patient selection for nerve‐sparing techniques, patients with localised PCa in the low‐ and intermediate‐risk groups and without evidence of tumour extension to the prostatic capsule or neurovascular bundles underwent bilateral nerve‐sparing procedures. Patients with high‐risk localised PCa or tumour extension involving only one side of the neurovascular bundles would be assessed for unilateral nerve‐sparing procedures on the side not involved by the tumour. Preoperative MRI scans were reviewed by radiologists to confirm the extent of tumour involvement. If the side initially planned for nerve sparing was highly suspicious for tumour involvement intraoperatively, the operator modified the nerve‐sparing approach accordingly. A ruler was introduced into the pelvis to measure the urethral stump available for vesicourethral anastomosis after removal of the prostate. The operating room setup and surgical manoeuvres are shown in Figure 1b and 2. The entire surgical procedure was videotaped and reviewed at the surgeon's request.

**FIGURE 2:**
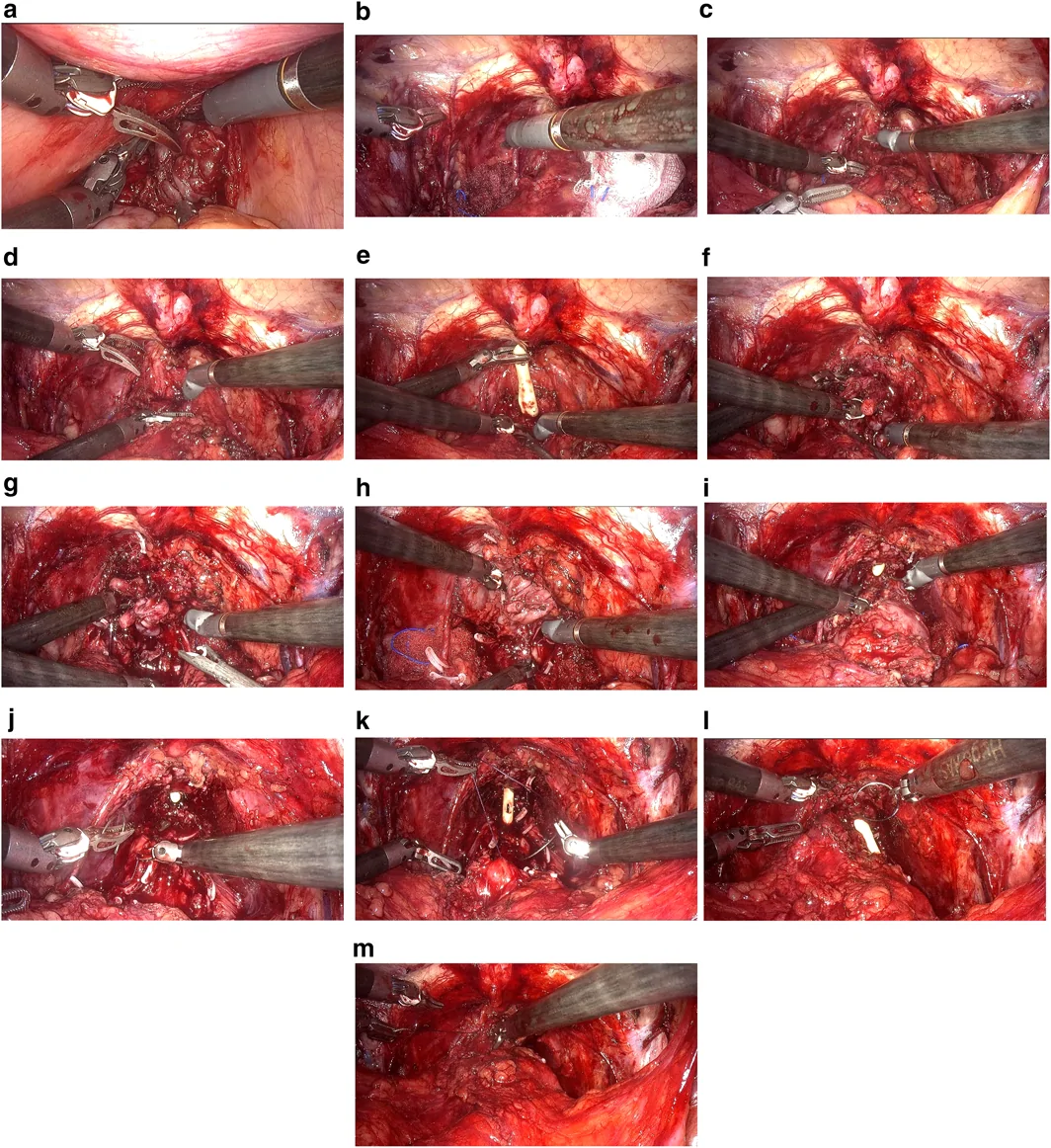
(a) Dissecting the seminal vesicles posteriorly from the cul‐de‐sac. (b) Entering the Retzius space and dissecting the bilateral obturator lymph nodes. The template included bilateral obturator fossae and the bilateral external iliac regions. (c) Clearing the preprostate fat. (d) Identifying the bladder neck by urethral catheter traction and incising the bladder neck using the monopolar scissor. (e) Keeping dissecting to the posterior aspect of prostate using upward traction of urethral catheter. (f) Pulling the seminal vesical anteriorly and totally dissecting the posterior aspect of prostate. (g) Dissecting the posterolateral aspect with help of the Hem‐o‐Lok (Teleflex, Wayne, PA, USA). (h) Totally dissecting the whole prostate except for the urethra and puboprostatic ligament. (i) Transecting the puboprostatic ligament and the urethral; dorsal vein ligation was not routinely performed in advance. (j) After taking down the prostate, careful suture ligation was done. (k) Adjusting the width of bladder neck before the vesicourethral anastomosis. (l) Performing the vesicourethral anastomosis using the 3‐0 V‐Loc (Medtronic plc., Minneapolis, MN, USA). (m) Completing the vesicourethral anastomosis.

The instructor, Yen‐Chuan Ou, MD, was in the operating room and ready to assist, sitting next to the operator (Figure 1c). His intervention was provided only at the operator's request. If the operator requested assistance, he would sit at the console and perform the surgical steps (Figure 1d). Console time began when the robotic arms were docked and the operator was seated at the console, ready to operate, and ended when the operator completed the final surgical step and undocked the robotic arms. The entire process was timed using the operator's iPhone (Apple Inc., Cupertino, California, USA). Blood loss was measured by recording the total volume in the suction bottle and subtracting the volume of normal saline used for irrigation. We then counted the number of surgical gauzes used during the procedure. Each gauze soaked with blood was considered to represent an additional 10 mL of blood loss. The console time and blood loss were recorded to assess the improvement of the operator after each case.

### 2.4 Perioperative Outcomes

The operator was responsible for the post‐operative hospital stays and clinical visits. The admission course was routinely scheduled for 7 days after RARP, and the urethral catheter was removed on the day of hospital discharge. Seven days after discharge, the first clinical visit will be conducted. The wound condition will be assessed, and bedside ultrasonography will be performed to evaluate the bilateral kidneys and bladder. Baseline pad use after urethral catheter removal will also be recorded. At 84–90 days and 174–180 days after RARP, the second and third clinical visits will be conducted, respectively, and PSA levels and pad use will be recorded. Any postoperative adverse events within 30 days were classified according to the Clavien–Dindo classification.

### 2.5 Oncological and Functional Outcomes

Positive surgical margins (PSMs) were reported with their size, location, and tumour Gleason grade group at the PSM site. A focal PSM was defined as a single unifocal lesion measuring less than 3 mm, whereas an extensive PSM was defined as multifocal lesions or a PSM measuring more than 3 mm. An undetectable PSA nadir level was defined as < 0.1 ng/mL according to the serum assay used at our institution. Because reaching the undetectable PSA nadir within 6 months was an independent predictor of biochemical recurrence‐free survival and distant metastasis‐free survival, we adopted achievement of the undetectable PSA nadir within 6 months as the oncological outcome [11]. PSA persistence was defined as failure to reach an undetectable PSA nadir after RARP. PSA recurrence was defined as a subsequent rise in PSA (> 0.2 ng/mL) from the undetectable PSA nadir after RARP. Late salvage radiation therapy would be applied if post‐operative PSA > 0.4 ng/ml. Recovery of continence was defined as using 0–1 pad per day after RARP. For ease of measure, the patients would be educated to use a certain brand of pad after discharge and record their daily use in the notebook after weighting.

### 2.6 Statistical Analysis

These data were recorded in Microsoft Excel (Microsoft Corp., Redmond, WA, USA). Analysis was carried out using R (R Core Team, 2024. R: Language and environment for statistical computing. R Foundation for Statistical Computing, Vienna, Austria. URL: https://www.R‐project.org/), and the ‘esvis’ package was used to perform the analysis. Nonparametric statistical methods were used for analysis and a type I error rate of 5% was applied.

## 3 Results

Fifteen men were recruited for this study. Baseline patient characteristics are summarised in Table 1, while intraoperative and postoperative parameters are presented in Table 2. Data distribution, outliers, and sensitivity analysis are reported in Table 3. The detailed console time and blood loss were estimated in Table 4. The median operative time (Supporting Information S1: Supplement 1), as abstracted from the nursing records, was 312 minutes (range: 245–784 minutes; interquartile range: 78 minutes). In Table 3, we also performed a sensitivity analysis. Based on the data distribution and the differences between the mean and 5% trimmed mean, blood loss appeared to be more robust than console time. Skewness and kurtosis indicated that outliers had a greater influence on the distribution of console time than on that of blood loss. A similar finding was observed when comparing the differences between the mean and 5% trimmed mean, further suggesting that console time was more susceptible to the influence of extreme values. In Table 4, both console time and blood loss showed a trend towards improvement in our first 15 cases.

**TABLE 1:** The general characteristics.

Age (years old); median (IQR^a^; [95% CI^b^])	70.50 (13.25; [64.33–75.13])
BMI^c^ (kg/m^2^); median (IQR^a^; [95% CI^b^])	22.00 (4.48; [20.33–24.22])
PSA (ng/ml); median (IQR^a^; [95% CI^b^])	40.40 (63.28; [20.02–87.65])
Total biopsy cores (*n*); median (IQR^a^; [95% CI^b^])	16.00 (4.00; [13.51–16.12])
Positive cores (*n*); median (IQR^a^; [95% CI^b^])	6.00 (3.00; [4.15–7.85])
Risk groups (*n*; %)	
Low	1 (6.67%)
Intermediate; favourable	2 (13.33%)
Intermediate; unfavourable	3 (20.00%)
High	4 (26.67%)
Very high	5 (33.33%)
Regional lymph nodes on MRI^d^ (*n*; %)	
Yes	5 (33.33%)
No	10 (66.67%)
Resected prostate weight (gm); median (IQR^a^; [95% CI^b^])	51.05 (24.35; [37.33–58.28])
Tumour percentage in resected prostate (*n*; %)	
Less than 5%	2 (13.33%)
More than 5%	13 (86.67%)
Pathological T stage (*n*; %)	
2a	1 (6.67%)
2b	2 (13.33%)
2c	2 (13.33%)
3a	5 (33.33%)
3b	5 (33.33%)
Pathological gleason group (*n*; %)	
2	3 (20%)
3	5 (33.33%)
4	5 (33.33%)
5	1 (6.67%)
Nerve‐sparing procedures	
Bilateral	9 (60%)
Unilateral	4 (26.67%)
Not performed	2 (13.33%)
Dissected lymph nodes (*n*); median (IQR^a^; [95% CI^b^])	9.00 (7.00; [5.91–10.64])
Lymph nodes involvement in surgical specimen (*n*; %)	
N0	12 (80%)
N1	3 (20%)

^a^ Interquartile Range.

^b^ Confidence Interval.

^c^ body mass index.

^d^ Magnetic Resonance Imaging.

**TABLE 2:** Intra‐ and post‐operative parameters.

Console time (min); median (IQR^a^; [95% CI^b^])	245.00 (78.00; [229.62–383.85])
Blood loss (ml); median (IQR^a^; [95% CI^b^])	400.00 (400.00; [320.97–552.36])
Positive surgical margin (*n*; %)	
No	12 (80%)
Yes	3 (20%)
Urethral stump used for urethrovesical anastomosis (cm); median (IQR^a^; [95% CI^b^])	3.10 (0.63; [2.89–3.40])
Continence rate (*n*; %)	
Before RARP^c^	15 (100%)
Baseline (1 week after discharge)	0 (0%)
3 months after RARP^c^ (*n*; %)	8 (53.3%)
6 months after RARP^c^ (*n*; %)	12 (80%)
PSA at postoperative 3 months (ng/ml); median (IQR^a^; [range])	0.33 (1.21; [0.01–10.30])
PSA at postoperative 6 months (ng/ml); median (IQR^a^; [range])	0.06 (0.25; [0.01–2.10])
Undetectable PSA nadir after 6 months (*n*; %)	
No	3 (20%)
Yes	12 (80%)

^a^ Interquartile Range.

^b^ Confidence Interval.

^c^ Robot‐assisted radical prostatectomy.

**TABLE 3:** The data distribution and outlier of (A) console time (median: 245 minutes) and (B) blood loss (median: 400 ml).

Variables	Skewness	Kurtosis	Mean	5% trimmed mean
Console time	2.24	4.74	306.73	290.26
Blood loss	0.15	−1.27	436.67	432.41

*Note:* The sensitivity analysis is provided at the bottom.

**TABLE 4:** The details of the console time and blood loss of each patient.

	Case 1	Case 2	Case 3	Case 4	Case 5	Case 6	Case 7	Case 8	Case 9	Case 10	Case 11	Case 12	Case 13	Case 14	Case 15
Console time (minutes)	543	367	309	244	234	275	203	226	245	231	707	223	256	303	235
Blood loss (ml)	700	800	550	600	400	375	200	200	200	150	500	250	375	650	600
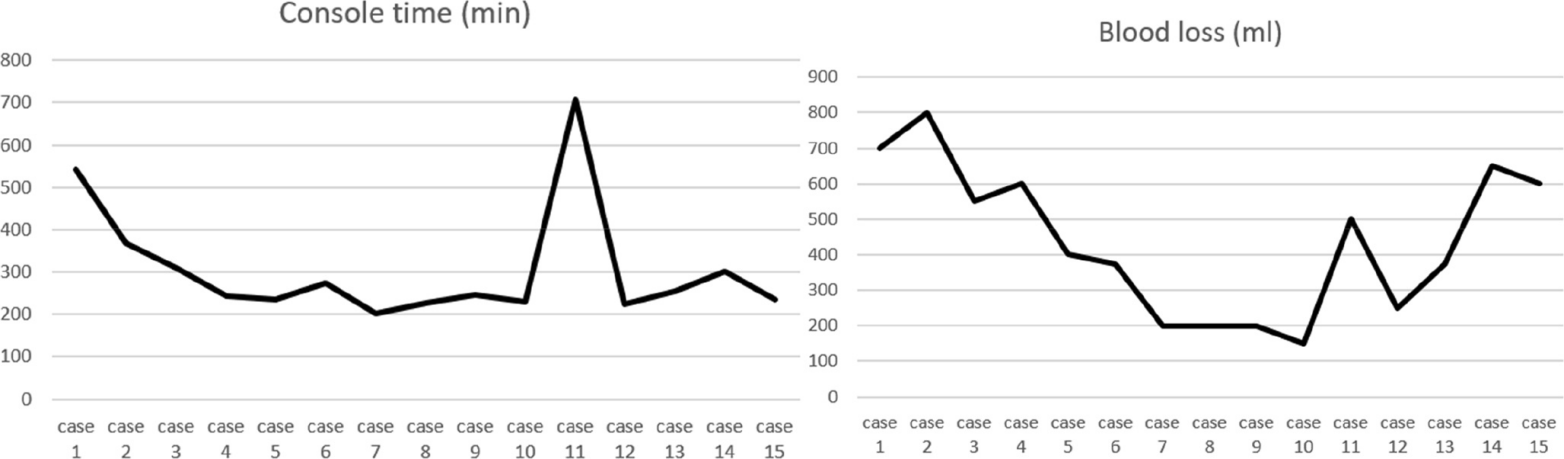															

In this cohort, more than 50% of patients had stage 3 disease at the time of surgery. The median weight of the resected prostate was 51 g. There were two men with previously metastatic PCa who underwent RARP after confirming that the cancer was controlled. Instructor assistance was provided only upon the operator's request and occurred exclusively during the first case. This initial case involved a patient with stage 3a disease and a preoperative PSA level of 23.5 ng/mL; the resected prostate weighed 54.6 g. The console time was 543 minutes, with an estimated blood loss of approximately 700 mL. Surgical steps requiring instructor intervention included bilateral obturator lymph node dissection (Figure 2b) and posterolateral dissection of the prostate (Figure 2f).

The 11th case involved a patient with morbid obesity (BMI 51.9 kg/m^2^) and a prior history of colon and rectal resection. Extensive adhesions were encountered intraoperatively, and the patient exhibited a narrow pelvic anatomy. The console time for this case was 707 minutes and resected prostate weighted 97 gm. Two days after RARP, decreased urine output was observed. Bedside ultrasonography showed that the balloon of the urethral catheter was located within the bladder, with no evidence of bilateral hydronephrosis. Three days after RARP, no urine output was observed from the urethral catheter, and bedside ultrasonography showed that the catheter balloon was no longer visible within the bladder. Non‐contrast computed tomography was performed and confirmed that the urethral catheter was located posterior to the bladder. Reoperation was performed, and intraoperatively, a rupture was identified in the posterior wall of the vesicourethral anastomosis. All sutures and debris surrounding the original anastomosis were removed, and a new anastomosis was performed (Figure 3). The total operative time was 184 minutes and the console time was 136 minutes. Since reoperation was required 3 days postoperatively in this case due to vesicourethral anastomotic rupture, this complication was categorised to be Clavien–Dindo grade IIIb. Besides the 11th case, only one postoperative complication was observed: pulmonary atelectasis on postoperative day 1, which prolonged the hospital stay by 3 days (Clavien–Dindo grade I). The overall complication rate was 13.33% (2/15), and the rate of significant complications (> Clavien–Dindo grade III) was 6.67% (1/15).

**FIGURE 3:**
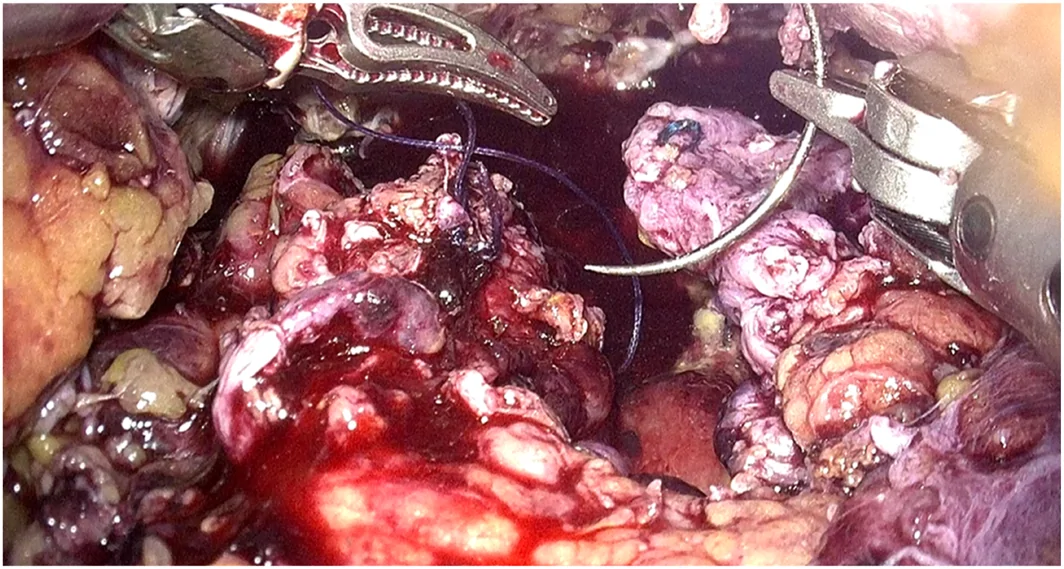
The vesicourethral anastomotic rupture and reoperation.

During the hospital stay, 13 men were discharged 7 days after RARP. One patient was discharged 10 days after RARP due to a postoperative Clavien–Dindo grade I complication, while another was discharged 22 days after RARP due to a postoperative Clavien–Dindo grade IIIb complication. All men had their urethral catheters successfully removed on the day of discharge.

Pathological staging revealed that 33.3% of patients had pT3a disease and 33.3% had pT3b disease. The median number of dissected lymph nodes during RARP was 9 (95% CI: 5.91–10.64). Twelve men (80%) had no lymph node involvement in the specimens, while three men (20%) were classified as pathological N1 stage. All of them had involved lymph nodes identified in the obturator regions. One patient had 2 involved lymph nodes out of 4 dissected lymph nodes in the right obturator region. Another had 1 involved lymph node out of 3 dissected lymph nodes in the right obturator region. The third had 2 involved lymph nodes out of 5 dissected lymph nodes in the right obturator region.

PSMs were observed in 20% of cases. In these three cases with PSMs, one had extensive PSMs involving the bilateral proximal and distal margins, bilateral peripheral margins, and bilateral cut ends of the vas deferens. The Gleason grade Group 4 was identified at the site of PSMs. Of the other two cases, one had a focal PSM at the apex, and the other had a focal PSM at the right posterolateral margin. Both of them had a Gleason grade group of 3 at the site of PSMs.

The median PSA level was 40.40 ng/mL (95% CI: 20.02–87.65) before RARP. At 3 months postoperatively, the median PSA level was 0.33 ng/mL (range: 0.01–10.30). At 6 months postoperatively, the median PSA level was 0.06 ng/mL (range: 0.01–2.01). Three patients (20%) failed to reach a postoperative undetectable PSA nadir within 6 months following RARP. Of these, one patient (6.67%) underwent late salvage radiotherapy, while the remaining two (13.33%) maintained PSA levels below 0.4 ng/mL.

None of the patients had incontinence before RARP. The median urethral stump length used for vesicourethral anastomosis was 3.1 cm. The baseline continence recovery rate at 1 week after discharge was 0%. Continence recovery at 3 months postoperatively was achieved in 8 patients (53.3%), while continence recovery at 6 months postoperatively was achieved in 12 patients (80%). Of the 3 patients who did not achieve continence recovery at 6 months after surgery, 1 had total incontinence and required 8–10 pads per day. Of the remaining 2 patients having stress incontinence, one of them required 3–4 pads per day, while the other required 2–3 pads per day.

## 4 Conclusion

In a previous publication regarding the first 30 cases performed using the DV system [12], the surgeon conducting RARP had experience with open RP (15–20 cases per year) and laparoscopic procedures. In this study, however, the surgeon was a first‐year attending physician with no prior experience in open RP and only experience as a first assistant in RARP. The recorded surgical video was reviewed only after the first case. Compared with the instructor [12], there were no prior D‐J stent insertions in our first 10 cases, and no ureteral orifice injuries occurred. In the instructor's first 15 cases, the maximal console time was 380 minutes [12]. Although the maximal console time was longer in this study, by the fourth RARP case, the console time, 244 minutes, in our study became comparable to that of the post–learning curve surgeon [13]. Similarly, the console times after the fourth case were comparable to those of the instructor's 16th to 30th cases [12]. In our first 15 cases, the console time demonstrated a trend towards improvement. A similar trend was observed in a surgeon following laparoscopic fellowship training [14], where the longest console time reached 480 minutes and the shortest was 200 minutes within the first 50 cases. In a previous publication, we concluded that surgeons familiar with the DV system might have quick adaptation to the Hugo RAS system [9]. Considering that the operating surgeon in this study had no prior experience with RP, we conclude that RARP using the Hugo RAS system is feasible in console time for a novice surgeon, even without previous open or laparoscopic RP experience.

Across published clinical trials, the 1‐year continence recovery rate was estimated at around 80%, while this rate of continence recovery could be achieved when performing nerve‐sparing (NS) technique [15, 16]. Among the studies currently available in the literature, the 3‐month continence recovery rate with the Hugo RAS system ranged from 75% to 100% [6], with the lowest rate reported by the surgical instructor of this study [9]. In this study, approximately 80% of patients in our first 15 cases achieved urinary continence and an undetectable PSA nadir within 6 months. One limitation of this study was that erectile function was not assessed using a validated questionnaire.

In our 11th case, we encountered the most severe complication, which required reoperation. The patient had a history of right hemicolectomy and was found to have severe adhesions in the cul‐de‐sac, along with massive scar tissue. The interval between TRUSP and surgery was only 2 weeks, and the tissue was fragile, with a tendency to ooze easily. In addition, the resected prostate volume was 97 g, meaning that most of the pelvic surgical field was occupied by the prostate. The patient was also extremely obese, with a narrow pelvic anatomy and a deviated urethral stump, which made vesicourethral anastomosis difficult. According to previous literature, the previous major abdominal surgeries such as hemicolectomy are risk factors for post‐RARP major complications, including anaemia and persistent drain output [17, 18]. A body mass index (BMI) over 35 kg/m^2^ is considered technically challenging [19]. Likewise, a prostate weighing more than 100 g is regarded as technically challenging. Although our resected prostate was 5 g lighter than the threshold for a technically challenging case, it was situated within a narrow pelvic anatomy [20]. Furthermore, the short interval between TRUSP and surgery remained a risk factor for complications in this case [21]. In this case, we learnt that careful case selection, proctoring, and structured training is essential for novice surgeons during the early stages of learning.

Our 12th case involved a patient with metastatic PCa who received triplet therapy [22, 23] and decided to undergo RARP after six cycles of treatment, with a PSA level < 0.2 ng/ml. Before surgery, a whole‐body bone scan showed regression of bone metastasis. This was actually the second case of RARP performed after achieving PSA control and metastasis regression in this scenario. Our fourth case received androgen deprivation therapy (ADT) for metastatic PCa and underwent RARP after confirming a PSA level < 0.2 ng/ml and complete regression of bone metastasis. After RARP, the postoperative PSA dropped to undetectable nadir (< 0.1 ng/ml) within 3 months. RARP was performed in these two cases because of the potential benefits including lower cancer‐specific mortality and overall survival [24, 25, 26, 27, 28, 29, 30]. In our experience, ADT administered before RARP can lead to shrinkage of the prostate and the adipose tissue surrounding the pelvic lymph nodes. The former may distort the anatomical planes, particularly when identifying the appropriate site for bladder neck incision. Novice surgeons should confirm the location of the prostate using multiple methods before incising the bladder neck. For example, we applied traction to the urethral catheter with the assistant while simultaneously compressing the urinary tract from the proximal bladder towards the distal urethra using two robotic arms. In addition to the bladder neck, the seminal vesicles may also become shrunken and fragile, requiring meticulous dissection. Regarding functional preservation, the plane between the prostatic bed and the neurovascular bundles may become more difficult to identify, and nerve‐sparing dissection should therefore be performed with great patience. For pelvic lymph node dissection, we observed in these two cases that although the reduced amount of adipose tissue provided a clearer operative field, the lymph nodes were more adherent to the obturator vessels and more prone to oozing. Novice surgeons should also exercise particular caution in this situation. In these two cases, PSA dropped to undetectable nadir at 6 months after surgery, and no perioperative complications occurred. All patients (100%) achieved R0 resection, and no lymph node involvement was identified in the specimens. However, one patient (50%) had not regained urinary continence at 6 months after RARP. In our experience, ADT before RARP increases anatomical distortion and creates additional technical challenges for novice surgeons. This finding is consistent with reports in the published literature [31, 32].

In our series of 15 cases, professional technical support staff from Medtronic were present throughout the procedures to provide assistance if any system‐generated customer messages (SGCMs) occurred [33]. Three types of SGCMs were encountered during the surgical procedures. The first type was robotic arm collision, which occurred in every case (100%). When this message appeared, the surgeon was required to dismiss the alert on the operator console to release the robotic arm from its safety freeze. The second type occurred when a robotic arm extended beyond the predefined surgical field. In these situations, the surgeon needed to reposition the camera arm and inspect the robotic arms at the patient bedside to exclude any potential collision and restore an adequate range of motion. This type of SGCM occurred in five (33.33%) cases that required more extensive pelvic lymph node dissection. Both of these SGCMs only required a brief interruption of the procedure and minor readjustments. Neither resulted in procedure abandonment, conversion to another surgical approach, or the need for a system power cycle. The third type of SGCM occurred in one (6.67%) case during vesicourethral anastomosis and was caused by failure of the internal cable within the needle driver arm. Inspection revealed that the instrument had exceeded its recommended service life and had been used beyond the manufacturer's specified number of uses. This event also did not result in procedure abandonment, conversion, or a system power cycle, and it was not attributable to operator error.

In previously published studies, we concluded that an experienced surgeon familiar with the DV system could quickly adapt to the Hugo RAS system [5, 9]. In this study of the initial 15 cases, we observed that a novice surgeon could adapt to the Hugo RAS system and achieve gradual improvement despite having no prior console experience with the DV system. In our experience, for the novice surgeons without prior console experiences, careful patient selection for those technically challenging cases, such as severe obesity, large prostate, previous major abdominal surgery, and locally advanced PCa, is still important to avoid the major complications and reoperation. Regarding the limitations, this study presents the experience of a single surgeon at a single institution; therefore, the outcomes may be influenced by differences in medical facilities and clinical settings. The number of cases was also limited, and the study may therefore have been statistically underpowered. Another limitation was that functional outcomes were not assessed using precise or standardized measures, which may have introduced bias into their evaluation. Regarding other sources of bias, our case selection was heterogeneous, with varying levels of case complexity. The data report indicated instructor assistance in the first case, and we excluded an outlier. These two factors constituted the major sources of bias in our data analysis. Without instructor assistance, the console time and estimated blood loss would have been greater than those reported. We included two cases of metastatic PCa after controlling disease progression; however, the surgical techniques differed from those used for RARP in patients with localised PCa. These technical differences may have introduced some degree of bias into the data. Based on the published literature, console time would be expected to be longer due to anatomical distortion, and blood loss would be expected to be greater due to an increased tendency for oozing. Regarding the exclusion of an outlier, although this decision was statistically justifiable, it may have made our findings less representative of real‐world scenarios. Differences in other variables, including BMI, prostate size, and disease stage, among cases may also have contributed to some degree of bias in our data.

Moreover, the follow‐up periods for both functional and oncological outcomes were relatively short, further limiting the interpretation and generalisability of the study findings. In the future, we will continue recruiting more first‐year surgeons, increasing the number of cases, and applying the system to a wider range of surgical categories. This article offers valuable insights into the initial supervised experience of the Hugo RAS system for a novice first‐year surgeon without prior console experiences. At the end of this article, we attached the current published literature and its novelty findings regarding the learning curve and surgical skills of RARP performed with the Hugo RAS system (Table 5) [34, 35, 36, 37, 38, 39, 40, 41, 42, 43]. In conclusion, Hugo RAS–assisted RARP was feasible during this supervised novice‐surgeon experience, with early adaptation in console time and blood loss. Based on our experience and the published literature [44], structured training may be beneficial in helping novice surgeons without prior console experience adapt more rapidly to performing individual or complete surgical procedures.

**TABLE 5:** The published studies regarding the learning curve and surgical skills of the Hugo RAS system.

Published literature	Findings
Marques‐monteiro et al. [34]	Offering the surgical setup of extraperitoneal robot‐assisted radical prostatectomy.
Veccia et al. [35]	New users can adopt the different docking angles.
Territo et al. [36]	Both intra‐ and extra‐ peritoneal approach are feasible with the hugo RAS system
Andrade et al. [37]	The major challenges for the new users are the docking position and bed assistant ergonomics
Dell'Oglio et al. [38]	The surgical setup of retzius‐sparing robot‐assisted radical prostatectomy with the hugo RAS system
Ou et al. [5]	The experienced users of the da vinci system can benefit with the quick adaption to the hugo RAS system
Ragavan et al. [10]	The operative time and docking time are similar between the da vinci system and the hugo RAS system
Antonelli et al. [39]	The hugo RAS system require more meticulous movement and lead to longer operative time than the da vinci system
Olsen [40]	Skills of the da vinci system can be transferred to the hugo RAS system
Ou et al. [9]	The intricate surgical steps would be lengthened, but the overall console time would not significantly change
Gandi et al. [41]	The proficiency in the da vinci system could be transferred to the hugo RAS system
Brime menendez et al. [42]	Compared with the da vinci system, the docking time is longer in the hugo RAS system but the operating time is similar. The times of bladder neck dissection and lymphadenectomy were longer in the hugo RAS system.
Ragusa [43]	The RARP could be performed with the hugo RAS system using three arms setting
This study	A novice surgeon could rapidly adapt to the hugo RAS system even without prior experience with the da vinci system.

## Author Contributions

**Che‐Hsueh Yang:** software, formal analysis, investigation, writing – original draft preparation, writing – review and editing, visualization. **Daniela‐Viviana Barbulescu:** conceptualisation, resources, supervision, project administration. **Lucian Marian:** methodology, investigation, writing – original draft preparation, visualisation. **Yuriy Aleksandrovich Kim:** methodology. **Chi‐Hsiang Wu:** validation. **Ragavan Narasimhan:** methodology. **Yen‐Chuan Ou:** resources. **Chi‐Chien Lin:** conceptualisation, methodology, data curation, writing – review and editing, supervision, project administration. All authors have read and agreed to the published version of the manuscript.

## Funding

The authors have nothing to report.

## Conflicts of Interest

The authors declare no conflicts of interest.

## Supporting information


Supporting Information S1

## Data Availability

The data analyzed in this retrospective study are subject to ethical and legal restrictions. De‐identified data may be made available from the corresponding author upon reasonable request and with the permission of the Institutional Review Board of Chang Bing Show Chwan Memorial Hospital.
