# Supplementary material for: Robot‐Assisted Radical Prostatectomy Using the Hugo RAS System: The First 15 Cases by a First‐Year Novice Surgeon Without Prior Console Experiences
Source: Int J Med Robot. 2026 Nov 2;22(6):e70249. doi: 10.1002/rcs.70249 (PMC13725931; doi:10.1002/rcs.70249)
Supplement: Supplementary file 1 [file RCS-22-e70249-s001.docx]

|  | Case 1 | Case 2 | Case 3 | Case 4 | Case 5 | Case 6 | Case 7 | Case 8 | Case 9 | Case 10 | Case 11 | Case 12 | Case 13 | Case 14 | Case 15 |
| --- | --- | --- | --- | --- | --- | --- | --- | --- | --- | --- | --- | --- | --- | --- | --- |
| Operation time (minutes) | 671 | 457 | 367 | 317 | 305 | 330 | 245 | 277 | 289 | 278 | 784 | 292 | 312 | 367 | 289 |

Supplement 1. The operative time of 15 cases.
